# Experimental analysis of oligonucleotide microarray design criteria to detect deletions by comparative genomic hybridization

**DOI:** 10.1186/1471-2164-9-497

**Published:** 2008-10-21

**Authors:** Stephane Flibotte, Donald G Moerman

**Affiliations:** 1 Canada's Michael Smith Genome Sciences Centre, BC Cancer Agency, Vancouver, B.C, V5Z 4S6, Canada; 2 Department of Zoology, University of British Columbia, Vancouver, B.C, V6T 1Z4, Canada; 3 Michael Smith Laboratories, University of British Columbia, Vancouver, B.C, V6T 1Z4, Canada

## Abstract

**Background:**

Microarray comparative genomic hybridization (CGH) is currently one of the most powerful techniques to measure DNA copy number in large genomes. In humans, microarray CGH is widely used to assess copy number variants in healthy individuals and copy number aberrations associated with various diseases, syndromes and disease susceptibility. In model organisms such as *Caenorhabditis elegans *(*C. elegans*) the technique has been applied to detect mutations, primarily deletions, in strains of interest. Although various constraints on oligonucleotide properties have been suggested to minimize non-specific hybridization and improve the data quality, there have been few experimental validations for CGH experiments. For genomic regions where strict design filters would limit the coverage it would also be useful to quantify the expected loss in data quality associated with relaxed design criteria.

**Results:**

We have quantified the effects of filtering various oligonucleotide properties by measuring the resolving power for detecting deletions in the human and *C. elegans *genomes using NimbleGen microarrays. Approximately twice as many oligonucleotides are typically required to be affected by a deletion in human DNA samples in order to achieve the same statistical confidence as one would observe for a deletion in *C. elegans*. Surprisingly, the ability to detect deletions strongly depends on the oligonucleotide 15-mer count, which is defined as the sum of the genomic frequency of all the constituent 15-mers within the oligonucleotide. A similarity level above 80% to non-target sequences over the length of the probe produces significant cross-hybridization. We recommend the use of a fairly large melting temperature window of up to 10°C, the elimination of repeat sequences, the elimination of homopolymers longer than 5 nucleotides, and a threshold of -1 kcal/mol on the oligonucleotide self-folding energy. We observed very little difference in data quality when varying the oligonucleotide length between 50 and 70, and even when using an isothermal design strategy.

**Conclusion:**

We have determined experimentally the effects of varying several key oligonucleotide microarray design criteria for detection of deletions in *C. elegans *and humans with NimbleGen's CGH technology. Our oligonucleotide design recommendations should be applicable for CGH analysis in most species.

## Background

In human health research microarray comparative genomic hybridization (CGH) has become a powerful technique to investigate DNA copy number variants (CNVs) in healthy subjects [1,2] and genomic aberrations associated with various diseases and syndromes [3,4]. Furthermore, CGH is now frequently used to analyze the genome of strains of interest in various model organisms [5,6]. On some oligonucleotide microarray platforms individual researchers can design their own specialized microarrays for very specific experiments. Basically, the only crucial requirement before starting to design an array is to have access to a sequenced reference genome for the species under investigation. The first task facing a biologist trying to design a CGH microarray is to design criteria to eliminate oligonucleotides with particular properties that are expected to reduce the data quality. Some design criteria have been suggested and used for several years with little or no large-scale experimental validation [7,8]. Large-scale studies of the effects of various oligonucleotide properties on microarray data quality are just starting to be published [9,10] but few of them are designed to investigate the two-colour scheme typically used in CGH experiments. Most of these studies are concerned with the human genome but it would be useful to know if some design criteria could be relaxed for smaller and less complex genomes and in general what kind of penalty one has to pay in terms of data quality when relaxing constraints on specific oligonucleotide properties.

In our research we are particularly interested in using oligonucleotide microarray CGH to detect induced deletions in the *C. elegans *genome [5,11]. We designed our own microarray chips but our criteria for oligonucleotide selection were arbitrary and relied more on empirical observation, that is the data quality was adequate for the task [5], and was not based on experimentally testing various oligonucleotide features. Optimal design criteria are expected to depend on the hybridization conditions and possibly on the complexity of the genome under investigation. In the current publication we report our findings on the effects of varying the oligonucleotide design criteria and how these alterations affect our ability to detect deletions in both the *C. elegans *and human genomes. Considering the differences in size and complexity of these two genomes the design properties we recommend here should be applicable to many organisms with a sequenced genome provided that the hybridization conditions are not drastically different from those used in our experiments.

## Results and discussion

### Effects of various oligonucleotide properties on resolving power

The concept of resolving power we use here was introduced in a software evaluation study [12]. It is a useful tool to detect and quantify small variations in overall data quality when changes are made to the oligonucleotide selection process or the data analysis procedure, or even when comparing different array platforms. Briefly, using the experimental distributions of the data points in the so-called normal regions and in the regions with copy number aberrations it is possible to estimate the expected p-value associated with the detection of a typical aberrant DNA segment covered by a given number of probes. In the current work, the resolving power in *C. elegans *has been evaluated with the help of two strains with large heterozygous deletions previously found in CGH experiments [5]. As a human sample, a pool of male DNA has been compared by CGH to a pool of female DNA so that probes targeting the X chromosome could be associated with a one-copy loss in the male sample. Details of the microarray design for both the human and *C. elegans *experiments can be found in the Methods section. Briefly, for both normal and deleted regions we had probes manufactured of length 50, 60, and 70 nucleotides. We also used a so-called isothermal design where the oligonucleotide length is varied in an attempt to obtain an approximately constant melting temperature. The only significant constraints applied on the oligonucleotides at the design stage were the exclusion of known repeats and for the human chip the elimination of segments with known CNVs and single nucleotide polymorphisms (SNPs). Microarrays with shorter oligonucleotides, for example 25-mers in the case of the Affymetrix platform [13], can also be used to infer CNVs [12,14] but their optimization [15,16] is associated with different issues than longer oligonucleotide arrays and therefore they will not be considered in the current study.

**Figure 1:**
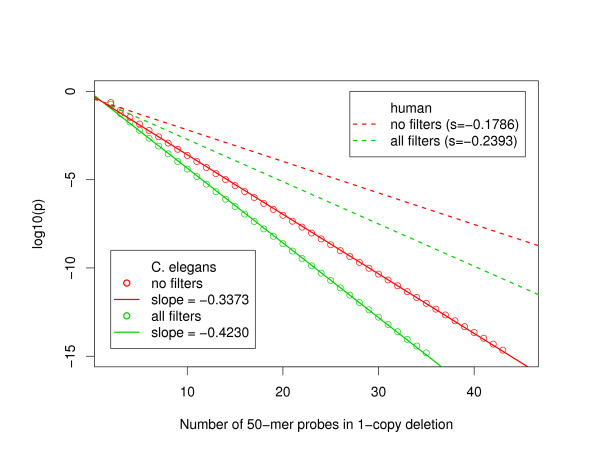
**Resolving power curves for detection of one-copy deletions with 50-mer oligonucleotides**. The open circles show the results from resolving power calculations for one-copy deletions, in other words, the logarithm of the expected p-value for a deletion is shown as a function of the number of probes affected by the copy-number aberration. The solid (dashed) lines are linear regressions of the resolving power calculations in *C. elegans *(human), with their slope being provided in the legends. Red data points and lines correspond to calculations using any oligonucleotide on the arrays without further filtering while the green lines and data points correspond to resolving power calculations after selecting the oligonucleotides with our standard filters as described in the Methods section.

Figure 1 shows resolving power curves for detection of one-copy deletions with 50-mer oligonucleotides for both *C. elegans *and human DNA with and without the application of standard constraints on the oligonucleotide properties. Those standard constraints are summarized in Table 1 and more details regarding their calculations can be found in the Methods section. It is clear that the resolving power curves are linear when plotted in logarithmic scale and therefore the data quality can be summarized by the slope, steeper slopes being better. For example, to achieve a p-value better than 1 × 10^-5 ^a typical heterozygous deletion in *C. elegans *would need to be covered by about 15 unfiltered oligonucleotides, while about 3 fewer probes would be required to achieve the same p-value if the standard filters are applied to the microarray design. The human data is noticeably noisier and one would basically require twice as many probes than in *C. elegans *to detect a deletion at a given p-value level. However, the improvement in the resolving power slope when applying the same standard filters is slightly better in the human example. More precisely, the ratio of resolving power slope between the filtered and unfiltered situations is 1.34 for human compare to 1.25 for *C. elegans*. However, those filters represent more restrictive design constraints for human DNA with only 24% of the 50-mer oligonucleotides on the array being accepted compare to 35% in the *C. elegans *case. It should however be noted that in the typical microarray designs we have used in previous biological experiments we did not apply a hard ceiling at the median value for the 15-mer count but simply used the 15-mer count to guide the final oligonucleotide selection for oligonucleotides passing all the other filters [5].

**Table 1:** Standard oligonucleotide filters used in this work.

**Oligonucleotide property**	**Condition for elimination**
Repeat sequences	Presence
Constituent 20-mers	Non-uniqueness
Homopolymers	Length > 5
Melting temperature	More than 5°C from median
Self-folding energy	< -1 kcal/mol
Similarity with other genomic location	> 70%
15-mer count	> median

**Figure 2:**
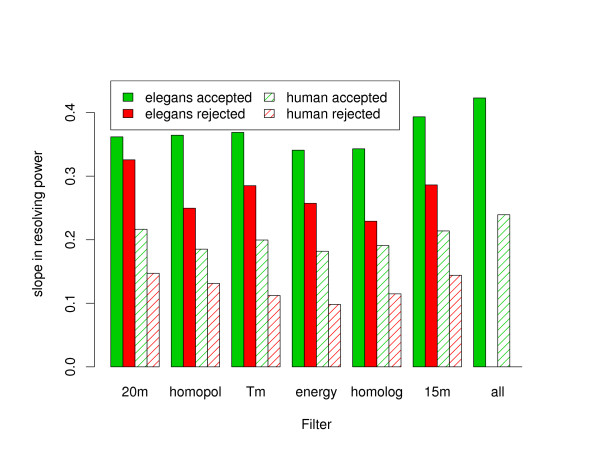
**Individual effect of standard oligonucleotide filters on resolving power**. The negative (or absolute value) of the slope in the resolving power curve is shown for individual constraints on oligonucleotides when detecting one-copy deletions with 50-mer probes. For each filter, the green bars correspond to resolving power calculations performed exclusively with oligonucleotides accepted by the filter while for the red bars only the oligonucleotides rejected by the filter were used in the calculations. Solid colours are associated with *C. elegans *and hashed areas are associated with the human data set. The filters from left to right are: elimination of non-unique 20 mers, elimination of homopolymers longer than 5 nucleotides, the selection of a 10°C range in melting temperature, the elimination of oligonucleotides with self-folding energy smaller than -1 kcal/mol, the elimination of oligonucleotides mapping to more than one genomic region with more than 70% similarity, the elimination of oligonucleotides with 15-mer count above the median value, and finally, the simultaneous application of all the those filters. More details on those standard filters can be found in the Methods section.

Each filter on oligonucleotide properties can be turned on and off independently before calculating the resolving power, except of course for the elimination of repeat sequences, which has been already applied to all the oligonucleotides present on the arrays. The effect of repeat sequences cannot be studied in the current work but this is not a significant limitation. It is true that in some cases it is possible to find sequences that are fairly unique within repeat regions, which might be of interest for some experiments especially in mammalian genomes [17]. However, at least in *C. elegans *we noticed that the CGH log2ratio signal tends to be somewhat unreliable with more non-zero bias near repeats. This type of alteration in signal is also often observed just outside deletions [5]. The individual effect of each of our oligonucleotide filters on resolving power is shown is Figure 2 for both human and *C. elegans *one-copy deletions detected using 50-mer oligonucleotides. The effect of each filter on the resolving power is virtually identical when using oligonucleotides of length 50, 60, 70, or with an isothermal design (data not shown). As previously mentioned, the slope in resolving power is roughly twice as steep for *C. elegans *than human data, and this is also true when individual filters are applied. For the most part, each filter produces a similar gain in resolving power for both human and *C. elegans *data except perhaps for the elimination of non-unique 20-mers which is more effective in human than *C. elegans*. However, the elimination of non-unique 20-mers is obviously a more restrictive design constraint in human than *C. elegans *as it eliminates 59% of all the oligonucleotides on the array compared to only 20% in the *C. elegans *case (data not shown).

**Figure 3:**
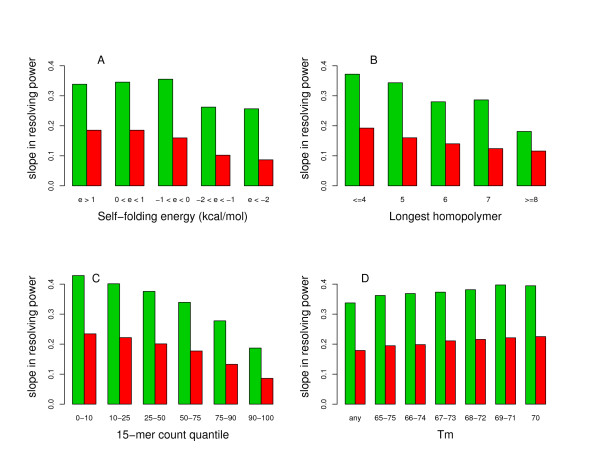
**Effects of varying some oligonucleotide constraints on resolving power**. The negative (or absolute value) of the slope in the resolving power curve is shown for individual constraints on oligonucleotides when detecting one-copy deletions with 50-mer probes. The green (red) bars correspond to *C. elegans *(human) data. The individual oligonucleotide constraints that have been varied consist of (A) the self-folding energy, (B) the length of the longest homopolymer, (C) the 15-mer count, and (D) the melting temperature.

It is of course possible to modify the parameters of some of our standard filters and measure the effect on the resolving power. Figure 3 shows the effects of changing the constraints on the self-folding energy, on the length of the longest homopolymer, on the 15-mer count and on the melting temperature both for human and *C. elegans *data obtained with 50-mer probes. Once again, the trends are very similar for human and *C. elegans *and for all the oligonucleotide lengths present on the arrays. As can be seen in panel A our standard use of a self-energy threshold of -1 kcal/mol seems optimal while panel B suggests that our standard ceiling of 5 for the longest homopolymer is certainly acceptable. In both human and *C. elegans *examples the vast majority of homopolymers are polyA and polyT tracts with much fewer polyC and polyG tracts so our results are basically measurements of the effect of having polyA and polyT present in a 50-mer probe. In contrast to what has been implied in a previous publication [9], the presence of polyA and polyT reduces the performance of our probes when attempting to detect deletions. However, since polyA/polyT tracts are much more frequent than polyC/polyG tracts, selecting probes with longer homopolymers tends to reduce the average GC content within the probes and therefore the average melting temperature. In order to disentangle the melting temperature and homopolymer effects we have performed another resolving power calculation but this time at a fixed melting temperature and our conclusion is still valid, the presence of long homopolymers tends to deteriorate the performance of a probe. As can be seen in panel C of Figure 3 filtering the probes according to their 15-mer count is a very efficient way to directly control their performance. For example, when calculating the ratio of the resolving power slopes for probes belonging to the bottom and top 10% in 15-count one obtains 2.3 for *C. elegans *and 2.7 for human. Finally, panel D demonstrates that our standard range of 10 degrees in melting temperature is an adequate filter and reducing the width of that window only marginally improve the data quality to detect deletions. This is in contradiction with what has been previously reported for general copy-number measurements in human samples [9]. As demonstrated in previous work [10], oligonucleotides with higher melting temperature tend to produce higher overall fluorescence intensities. However, the use of a two-colour CGH scheme coupled with our data analysis procedure appears to eliminate the need for a very uniform melting temperature design. The formula we used to calculate the melting temperature is identical to that used in Reference [9] with only a small difference in one of the parameters, which cannot affect our conclusion for oligonucleotides of fixed length. We have repeated the resolving power analysis but this time with a melting temperature calculation based on a nearest neighbour approach [18-20] and once again we see only a marginal improvement in data quality when reducing the width of the window in melting temperature (data not shown).

**Figure 4:**
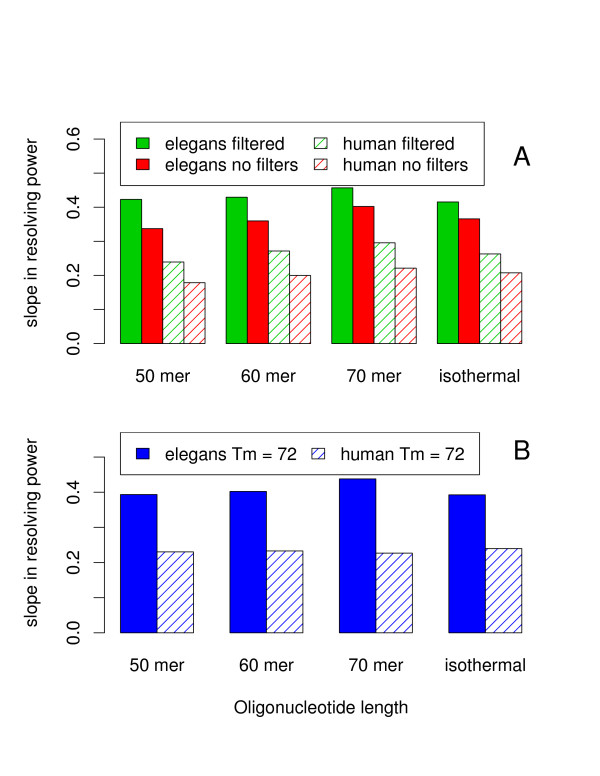
**Effects of varying the oligonucleotide length**. The negative (or absolute value) of the slope in the resolving power curve is shown as a function of oligonucleotide length for C. elegans (solid colour bars) and human (hashed bars). (A) Effects of varying the oligonucleotide length between 50 and 70 before (red bars) and after (green bars) the use of our standard filters, see Methods for details. For the so-called isothermal design the length of each oligonucleotide was allowed to vary between 50 and 70 in an attempt to minimize the width to the melting temperature distribution. (B) Effects of varying the oligonucleotide length between 50 and 70 for a fixed melting temperature of 72°C without applying additional constraints on oligonucleotides.

As previously mentioned and illustrated in Figure 4 Panel A, the trends we observed for the resolving power are very similar for oligonucleotides of length 50, 60, 70 and our isothermal design. A very small gain in performance is observed for longer probes but as can be deduced from panel B of Figure 4 the majority of that gain is probably due to the fact that longer probes tends to have higher melting temperature.

### Sequence similarity

In order to quantify the best design practices with regard to minimizing potential non-specific hybridization we have introduced a series of perturbations on a pre-selected number of oligonucleotides, see Methods section for details. Basically, two different kinds of sequence similarity have to be considered, either the presence of a stretch of perfect identity of a given length within the oligonucleotide or a given similarity level over the whole oligonucleotide.

**Figure 5:**
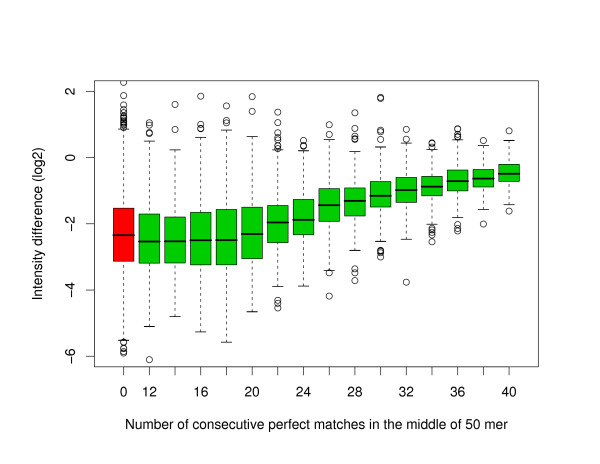
**Stretch of perfect identity in the middle of 50-mer oligonucleotides in *C. elegans***. Boxplots of the difference in fluorescence intensity in log2 scale between the original and perturbed 50-mer oligonucleotides. For the green boxplots, the perturbation consisted in randomizing the left and right sides of the original oligonucleotide while keeping a stretch intact in the middle. The red boxplot is associated with a randomization over the full length of the oligonucleotide. In all the cases, the perturbed oligonucleotide has the same GC content as the original oligonucleotide in an attempt to keep the melting temperature constant.

**Figure 6:**
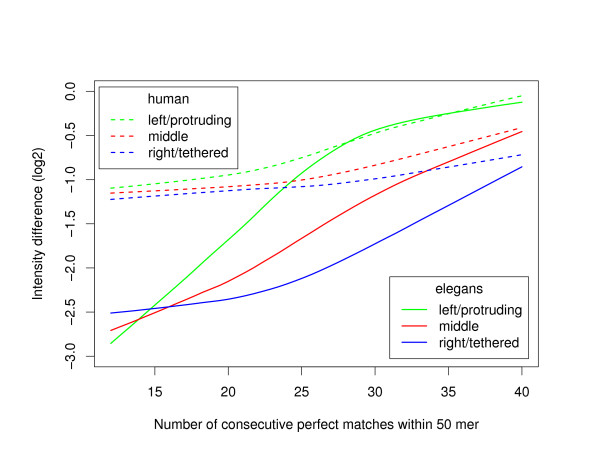
**Effect of the position of a stretch of perfect identity within 50-mer oligonucleotides**. LOESS regression of the difference in fluorescence intensity (in log2 scale) between the original and perturbed 50-mer oligonucleotides as a function of the length of the stretch of perfect identity. Solid (dashed) lines correspond to *C. elegans *(human) data. The perfect stretch of identity is either on the left (5') side (green lines), right (3') side (blue lines) or middle (red lines) of the 50-mer oligonucleotide. With NimbleGen's manufacturing process the oligonucleotides are synthesized from 3' to 5' and therefore the left side is protruding and freely floating in the solution while the right side is closer to the slide.

The red boxplot in Figure 5 shows the difference in fluorescence intensity one is expected to observe between a 50-mer oligonucleotide mapping perfectly to the *C. elegans *genome and a random 50-mer oligonucleotide with the same GC content. This is the basis for comparison and oligonucleotides with sequence identity associated with a smaller difference in intensity present some level of cross-hybridization. As can be seen from the green boxplots in Figure 5, a stretch of perfect identity of length of about 22 and above in the middle of the oligonucleotide will produce some level of cross-hybridization, and of course the longer the perfectly matched sequence is the worst the effect will be on the performance of the oligonucleotide. The elimination of non-unique 20 mers in our standard filters seems therefore a little too conservative. However, as can be seen in Figure 6, the position of the stretch of perfect identity within the oligonucleotide is important. Presumably due to steric effects, a stretch of perfect identity close to the slide will produce less cross-hybridization problems than a perfect stretch of identical length located at the other end of the oligonucleotide. For example, in *C. elegans *a perfect match of length 30 in the middle of the oligonucleotide will introduce similar cross-hybridization noise as a perfect match of length 23 close to the slide or length 36 at the end away from the slide. In fact, a perfect match of length 20 at the end away from the slide will produce a measurable fluorescence intensity above background so our standard elimination of non-unique 20 mers is justifiable in these instances. Similar positional effects are manifest in our human data set, except that the overall amplitude of the intensity difference between original and perturbed oligonucleotides is smaller. This is because the human data is noisier and spans a smaller dynamical range. This effect is compatible with the asymmetry previously reported for experiments performed with one-colour hybridization scheme on NimbleGen microarrays [10]. Furthermore, such asymmetry could explain the difference in performance sometimes observed [21] between oligonucleotides designed following the plus and minus strand templates at a given genomic location.

**Figure 7:**
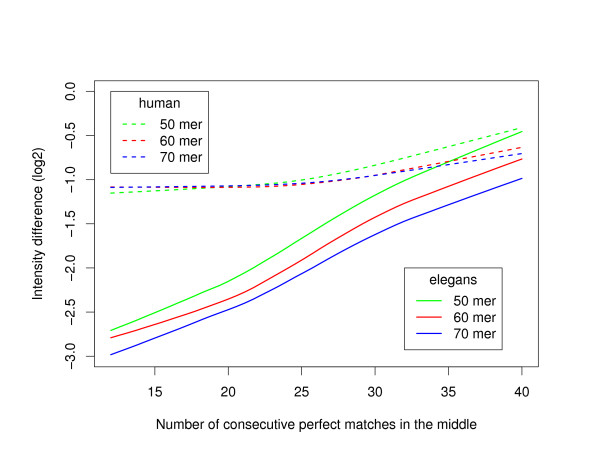
**Stretch of perfect identity in the middle of oligonucleotides of various lengths**. LOESS regression of the difference in fluorescence intensity (in log2 scale) between the original and perturbed oligonucleotides as a function of the length of the stretch of perfect identity. Solid (dashed) lines correspond to *C. elegans *(human) data. Results are shown for oligonucleotides of length 50 (green lines), 60 (red lines) and 70 (blue lines).

As expected, for *C. elegans *the presence of a perfect stretch of identity of a given length will produce a higher level of cross-hybridization for shorter oligonucleotides. For example, as can be seen in Figure 7, a perfect stretch of length 30 in the middle of a 60-mer oligonucleotide will produce the same intensity perturbation as a perfect stretch of length 27 in the middle of a 50-mer oligonucleotide, or a perfect stretch of length 33 in the middle of a 70-mer oligonucleotide. Figure 7 also shows that such a trend is not quite as obvious in the human case, in particular, very little difference is seen between the curves for 60- and 70-mer oligonucleotides. It should be noted that the recommendation [7] of eliminating non-unique 15 mers within oligonucleotides of length 50 is too conservative with our hybridization conditions. This is fortunate because basically no oligonucleotides would pass such a constraint for CGH in large genomes. However, as already mentioned, minimizing the 15-mer count of oligonucleotides is recommended to improve the resolving power.

**Figure 8:**
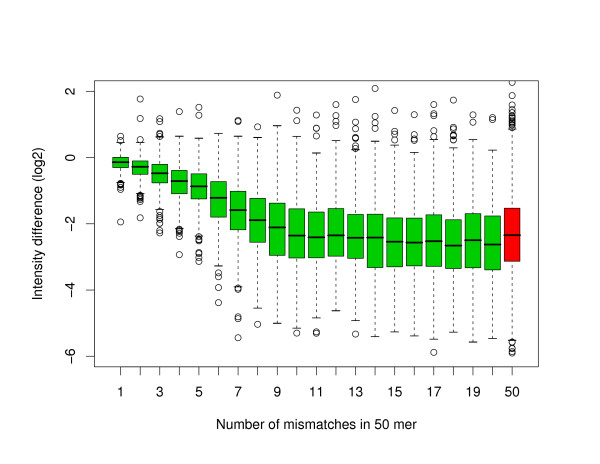
**Introduction of random mismatches in 50-mer oligonucleotides in *C. elegans***. Boxplots of the difference in fluorescence intensity in log2 scale between the original and perturbed 50-mer oligonucleotides. For the green boxplots, the perturbation consisted in the introduction of mismatches at random locations. The red boxplot is associated with a randomization over the full length of the oligonucleotide. In all the cases, the perturbed oligonucleotide has the same GC content as the original oligonucleotide in an attempt to keep the melting temperature constant.

**Figure 9:**
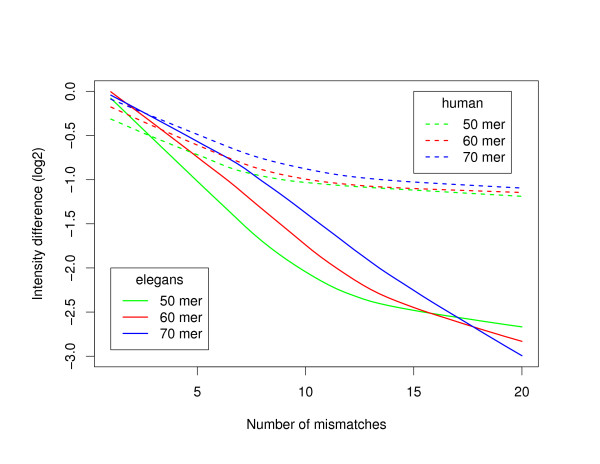
**Introduction of random mismatches in oligonucleotides of various lengths**. LOESS regression of the difference in fluorescence intensity (in log2 scale) between the original and perturbed oligonucleotides as a function of the number of mismatches introduced in the oligonucleotide. Solid (dashed) lines correspond to *C. elegans *(human) data. Results are shown for oligonucleotides of length 50 (green lines), 60 (red lines) and 70 (blue lines).

Figure 8 shows that the introduction of about 10 or more mismatches within a 50-mer oligonucleotide is enough to bring the fluorescence intensity down to the background level in *C. elegans*. One can see in Figure 9 that the corresponding limits for 60-mer and 70-mer oligonucleotide are about 12 and 14 mismatches, respectively. In other words, an oligonucleotide mapping to a second location in the genome with an overall degree of similarity above about 80% will produce a measurable amount of non-specific hybridization, which is in agreement with what has been reported in previous work for 50-mer oligonucleotides [7]. When comparing with the example used above for a stretch of perfect identity of length 30 in the middle of a 50-mer oligonucleotide, one can see that the same level of cross-hybridization would be obtained for an oligonucleotide with an overall similarity around 88% over the length of the oligonucleotide. Once again, the smaller dynamical range covered by the human data (see dashed lines in Figure 9) makes a precise interpretation of the results more difficulty. However, even in the human case, it is clear that for a fixed number of mismatches the shorter oligonucleotides will present more perturbation on the fluorescence intensity.

### Oligonucleotide design recommendations

Several constraints can be applied on oligonucleotide properties to improve the data quality but one should keep in mind that our design recommendations summarized in this section could easily be relaxed to improve coverage in specific genomic regions and still get useful information from the resulting data. In the current work we have only studied one-copy deletions but we expect that oligonucleotide design criteria improving the resolving power for detection of deletions should also improve the resolving power for detecting copy number gains. However, we cannot really infer that our design recommendations are necessarily optimal for experiments attempting to measure precise copy numbers where large copy numbers are expected.

We observed very little difference in data quality when varying the oligonucleotide length between 50, 60, 70, and even when using a so-called isothermal design where the length of each oligonucleotide varies between 50 and 70 in an attempt to minimize the overall width of the melting temperature distribution. Oligonucleotides of fixed length are simpler to work with at the design stage and it is easier to avoid unwanted genomic regions with shorter oligonucleotides. Even if only for convenience we will continue to use 50-mer oligonucleotides in our own research projects and suggest that oligonucleotides of this length will suffice for most other projects.

Our results demonstrate that filtering potential oligonucleotide probes according to their 15-mer count is probably the most effective way to control probe quality. In the current work repeat sequences have been eliminated right from the start and we therefore do not have a direct measurement of their effect on data quality. However, considering the effect of the 15-mer count above it is safe to assume that most types of repeats should be excluded from most CGH array designs. The quality of the data can be improved by considering the oligonucleotides self-folding tendency and the presence of homopolymers; a self-energy threshold of -1 kcal/mol seems optimal and the elimination of oligonucleotides with homopolymers longer than 5 is probably adequate in most situations. Only small gain in data quality is achieved by restricting the oligonucleotides melting temperature and using a relatively wide window of 10°C centered on the median value seems an acceptable compromise between data quality and coverage.

Two types of sequence similarity with multiple genomic regions have been investigated, the presence of a perfect identity over a fraction of the probe and the similarity over the whole length of the probe. Our elimination of non-unique 20 mers within the genome when designing oligonucleotides is conservative and is really only justified when the 20-mer is located at the end away from the slide in a 50-mer probe. A stretch of perfect identity of length 22 in the middle of a 50-mer probe will produce measurable cross-hybridization. The same is true for a similarity level above about 80% over the full length of a probe. While the constraints on oligonucleotide design described here are good starting points, the optimal constraints to be used to eliminate cross-hybridization from both types of sequence similarity will depend on the genome under investigation and the desired coverage in a given region.

## Conclusion

We have analyzed CGH experiments performed with NimbleGen's microarray platform in order to assess the relationships between various oligonucleotide properties and the quality of the data as measured by the ability to detect deletions in the human and *C. elegans *genomes. For the most part our microarray design recommendations summarized in the previous section are very similar for both species and they could probably be used without modifications for most other species with a sequenced reference genome. As expected, the larger and more complex human genome is more difficult to study with CGH and a deletion typically needs to affect approximately twice as many probes to achieve the same level of statistical confidence as in *C. elegans*. All our results were obtained with the NimbleGen platform with their standard hybridization protocol and of course our conclusions might not be valid for other microarray platforms or when using different hybridization conditions.

## Methods

### DNA

DNA from two *C. elegans *strains harbouring deletions were used as samples in the current study; strains VC10019 (gk487/mIn1) and VC10020 (gk488/mIn1) [5] carry 0.8 Mb and 0.5 Mb heterozygous deletions on chromosome II, respectively. For both *C. elegans *hybridizations, DNA extracted from the wild-type N2 strain has been used as the reference DNA. For the human DNA experiment, the sample was a commercial pool of DNA from 6 male anonymous individuals and the reference was a similar DNA pool from 6 female donors both supplied by Promega Corporation. Details of the nematode culture, DNA preparation and labelling can be found in a previous publication [5].

### Oligonucleotide microarray design

Both the human and *C. elegans *microarrays used in the current study comprised approximately 380 × 10^3 ^oligonucleotides tiling the positive strand. In each case the total number of oligonucleotides was divided in five approximately equal parts: 1) oligonucleotides of length 50, 2) oligonucleotides of length 60, 3) oligonucleotides of length 70, 4) oligonucleotides of variable length between 50 and 70 selected to minimize the overall spread in melting temperature, and finally 5) perturbed oligonucleotides where mismatches have been intentionally introduced in oligonucleotides from the first three categories above. The last category has been subdivided in three types of perturbation 1) a complete random shuffling, 2) the introduction of mismatches at random locations or 3) a random shuffling of the nucleotides at one or both ends in order to produce oligonucleotides with perfect matches on the left, right or middle. Each perturbed oligonucleotide originated from a perfect match oligonucleotide passing our standard constraints, see below. Furthermore, the GC content of the perturbed oligonucleotide was identical to the GC content of the original oligonucleotide in an attempt to maintain the same melting temperature. Each perturbed oligonucleotide can therefore be associated with a specific perfect match oligonucleotide present on the array and the difference in fluorescence intensity between the pair should be a reflection of the perturbation applied. The number of oligonucleotides in each category is provided in Table 2 for the *C. elegans *and human arrays.

**Table 2:** Number of oligonucleotides for each category represented on the arrays.

**Species**	**Oligonucleotide Length**	**Perfect Match**	**Perturbed oligonucleotides**		
			Random Shuffling	Random Mismatches	Perfect Stretch
*C. elegans*	50	76305	3815	9854	7618
	60	76463	3814	16140	12468
	70	75223	2232	10714	6431
	Isothermal	75439	3586	0	0
Human	50	75769	3171	10268	8294
	60	75784	3166	14448	11658
	70	75805	1841	13344	8280
	Isothermal	75530	2812	0	0

For the *C. elegans *array design, the oligonucleotides selected correspond to an approximately uniform tiling of the deletions in gk487 and gk488 plus 0.5 Mb of flanking regions on each side. The repeats annotated in Wormbase data freeze version WS170 have been eliminated from consideration but no other constraints have been applied on the oligonucleotides except that they had to be synthesized in fewer than 180 cycles with NimbleGen's microarray manufacturing process [22]. Approximately 22% and 16% of the oligonucleotides cover the deletions in gk487 and gk488, respectively.

A similar strategy has been applied to select the oligonucleotide for the human array. In this case the probes were approximately uniformly distributed on the whole genome but with increased density for chromosome X resulting in approximately 37% of the oligonucleotides covering that chromosome. Once again, repeats were eliminated but also the regions with known SNPs (in dbSNP) [23], CNVs and other genomic variants (in the Database of Genomic Variants) [24,25].

### Hybridization and data processing

The hybridization, image analysis, extraction of fluorescence intensities and their ratios log2ratio together with their subsequent normalization have been described in detail in a previous publication [5]. Briefly, a two-colour CGH scheme has been used and the hybridization and image analysis have been performed as a commercial service by Roche NimbleGen Inc. No background has been subtracted before calculating the log2ratio values and the normalization followed a LOESS regression. The data discussed in this publication have been deposited in NCBI's Gene Expression Omnibus [26] and are accessible through GEO Series accession number GSE12208 [27].

### Resolving power

The concept of resolving power, as used in the current work, has been described in a previous publication [12]. The inputs to the resolving power calculations are the mean and standard deviation of the log2ratio data points in the normal and aberrant regions. Consequently, the calculations assume that the distribution of log2ratio is Gaussian for both types of region and no attempt is made to account for possible autocorrelation between data points mapping to nearby genomic locations. Armed with the mean and standard deviation of both distributions and knowing the total number of data points on the array one can evaluate the expected p-value for copy number aberrations affecting a given number of probes with the same mathematical formulation that is used to calculate a t-test. In the current study we are only interested in calculating the resolving power for one-copy deletions. The logarithm of the p-value coming out of a resolving power calculation is linear with the number of probes affected by the copy-number aberration and therefore a resolving power curve can be summarized by its slope, which is easily calculated with a linear regression.

### Oligonucleotide properties and standard filters

We are using the term standard filters to refer to the microarray design constraints on oligonucleotides similar to those that gave us acceptable results in previous CGH studies [5]. In summary they correspond to 1) the elimination of repeat sequences, 2) the elimination of 20-mers occurring more than once in the genome, 3) the elimination of homopolymers longer than 5 nucleotides, 4) the selection of oligonucleotides with a melting temperature *T*_*m *_within +- 5°C of the median melting temperature where *T*_*m *_has simply been calculated as a function of percent GC content and oligonucleotide length *L *by *T*_*m *_= 64.9 + 0.41GC - 500/*L*, 5) the elimination of oligonucleotides with a self-folding energy smaller than -1 kcal/mol according to a hybrid-ss-min calculation [28], 6) the elimination of oligonucleotides mapping to more than one location in the genome with a similarity level above 70% over the whole oligonucleotide according to a MegaBLAST [29] search, and 7) the elimination of oligonucleotides with a 15-mer count above median where the 15-mer count is defined as the sum of the genomic frequency of all the constituent 15-mers within the oligonucleotide. With the exception of the first constraint, all the filters can be modified at the analysis stage before calculating the resolving power, the repeats have already been eliminated when designing the arrays and therefore that constraint cannot be modified during the analysis and applies to all the results presented in the current work.

## Authors' contributions

SF designed the study and the microarrays, performed the data analysis and drafted the manuscript. DGM helped to draft the manuscript. Both authors read and approved the final manuscript.
